# Adsorption characteristics of Er_3_N@C_80_on W(110) and Au(111) studied via scanning tunneling microscopy and spectroscopy

**DOI:** 10.3762/bjnano.8.114

**Published:** 2017-05-23

**Authors:** Sebastian Schimmel, Zhixiang Sun, Danny Baumann, Denis Krylov, Nataliya Samoylova, Alexey Popov, Bernd Büchner, Christian Hess

**Affiliations:** 1 Leibniz-Institute for Solid State and Materials Research, IFW-Dresden, Helmholtzstrasse 20, 01069 Dresden, Germany; 2 Institut für Festkörperphysik, TU Dresden, 01069 Dresden, Germany; 3 Center for Transport and Devices, TU Dresden, 01069 Dresden, Germany

**Keywords:** adsorption, Au(111), Er_3_N@C_80_, scanning tunnelling microscopy, scanning tunnelling spectroscopy, W(110)

## Abstract

We performed a study on the fundamental adsorption characteristics of Er_3_N@C_80_ deposited on W(110) and Au(111) via room temperature scanning tunneling microscopy and spectroscopy. Adsorbed on W(110), a comparatively strong bond to the endohedral fullerenes inhibited the formation of ordered monolayer islands. In contrast, the Au(111)-surface provides a sufficiently high mobility for the molecules to arrange in monolayer islands after annealing. Interestingly, the fullerenes modify the herringbone reconstruction indicating that the molecule–substrate interaction is of considerable extent. Investigations concerning the electronic structure of Er_3_N@C_80_/Au(111) reveals spatial variations dependent on the termination of the Au(111) at the interface.

## Introduction

Fullerenes provide the feasibility of tunable physical properties by their capacity to encapsulate atoms or clusters inside the carbon cage [1–2]. Thus since their discovery in 1985 they excite great attention of the scientific community. Sustained efforts on the synthesis of endohedral fullerenes led to the trimetallic nitride template (TNT) process and consequently to the creation of the class of trimetallic nitride endohedral fullerenes in 1999, which can be produced in a sufficiently high yield for experimental studies and possible applications [3]. These molecules A_3−_*_n_*B*_n_*N@C*_k_* (*n* = 0–3; A, B = rare earth metal or transition metals of the IVth subgroup; *k* = 68–96) are composed of a carbon cage which encapsulates a triangular cluster consisting of 3 rare-earth or transition metal atoms and a nitrogen atom at its center [4]. Dependent on the cluster composition and due to the intercalation inside a protecting carbon cage, intriguing properties emerge. For instance, single molecular magnetism was observed for DySc_2_N@C_80_ [5] and conductance switching by tunneling current induced cluster rotations between chiral conformations was demonstrated for Sc_3_N@C_80_ [6]. Furthermore, the magneto-optically active endohedral fullerene Er_3_N@C_80_ permits a direct non-cage-mediated optical interaction with the incarcerated Er^3+^ ion in near-infrared that might make its use as optical manipulable fullerene-qubit possible [7–9]. Due to the versatile characteristics, the trimetallic nitride endohedral fullerenes are considered as promising candidates for applications in the fields of molecular electronics, molecular spintronics and quantum information processing. The implementation of these ambitious applications requires the knowledge about the molecules’ behavior in interaction with possible electrode surfaces. One aspect regards the formation of one respectively two dimensional and addressable arrays. Another important issue concerns the elucidation of the system’s electronic structure and adsorption site dependent effects on it.

In order to examine the adsorption characteristics and the electronic structure of Er_3_N@C_80_ in consideration of adsorbate–substrate interaction, we performed scanning tunneling microscopy (STM) and scanning tunneling spectroscopy (STS) investigations on sub-monolayer covered W(110) and Au(111) single crystal substrates. Beside their potential application as electrode materials, the choice of these established standard substrate for STM/STS investigations provides the advantages of comparability to results of earlier measurements and well-known fast cleaning treatments.

## Experimental

Er_3_N@C_80_ was purchased from SES Research. For STM measurements, the samples was purified by high-pressure liquid chromatography (HPLC) with Buckyprep-M column and toluene as a solvent, washed with acetone and hexane, and then transferred to the crucible of the Knudsen cell by drop-casting from toluene.

To achieve the reproducible preparation of sub-monolayer Er_3_N@C_80_-coverage on substrates with the demanded cleanliness for systematically STM/STS investigations, the molecules were deposited via organic molecular beam epitaxy under ultra-high vacuum (UHV) conditions (*p* < 10^−9^ mbar) and subsequently analyzed in situ in a variable temperature STM.

To provide contamination free substrate surfaces, cleaning treatments were applied to the used single crystals, prior to the measurement. According to the proceeding suggested by Bode et al. [10], the W(110)-surface was cleaned by repeated cycles of annealing (*T* ≈ 1500 K) at increased oxygen pressure and subsequent e-beam flashing (*T* ≈ 2300 K). This process was conducted 4 times in which the oxygen raised chamber pressure was stepwise reduced from *p* ≈ 5 × 10^−7^ mbar down to *p* ≈ 2 × 10^−8^ mbar. Thus, the W(110)-surface possesses clean terraces of monoatomic height and without its conventionally carbon-induced reconstruction. The preparation of the Au(111)-surface was done by Ar-ion sputtering with an ion-energy of 1 keV. By posterior annealing (*T* ≈ 823 K; *t* ≈ 60–120 min) extended terraces with monoatomic step edges could be obtained. By this standard procedure, as is typical for a clean Au(111) surface, the herring bone reconstruction occurred. The success of these prior treatments was checked by STM before depositing the molecules.

After that the thermally stable endohedral fullerenes Er_3_N@C_80_ were evaporated from a home-built and carefully degassed evaporator for *t* ≈ 4 min at a temperature of about *T* ≈ 800 K on the single crystal substrates. By this procedure, samples with a coverage of about 16% could be prepared reproducibly. In order to induce the formation of monolayer height molecule islands on the surfaces, the substrates were heated (*T* = 620–670 K) during and after the deposition, to increase the molecule mobility [11–12]. This annealing treatment was applied to all samples presented in this study.

The measurement data was acquired at room temperature and under UHV conditions. The topographic images were produced in constant-current mode, with the bias voltage applied to the tip. The generated images were processed using WSxM [13]. The spatially resolved spectroscopy information was taken by *I*(*U*) measurements at open feedback loop at every pixel of the corresponding image. In order to obtain d*I/*d*U*(*U*) data a posterior numerical derivation using the analysis software WSxM [13] was performed. For the measurements, mechanically cut Pt–Ir-tips have been used.

## Results and Discussion

**Figure 1:**
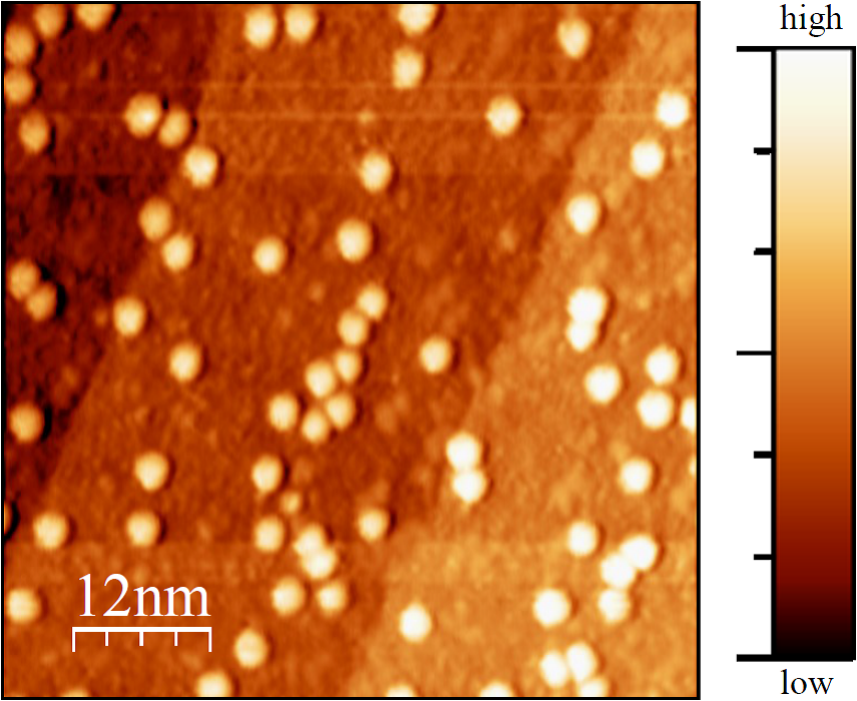
Topographic image (*U* = 2 V; *I* = 0.5 nA) of Er_3_N@C_80_ on W(110). The molecules appear as bright round structures on the straight monoatomic steps of W(110). The fullerenes are randomly distributed on the surface. The residual roughness visible in the image most probably emerges from multiple imaging of the molecules due to the imperfection of the tip. The scale bar colors are also representative for all following images.

Following the previously mentioned treatments for depositing Er_3_N@C_80_, on clean W(110) sub-monolayer-coverage could be obtained as verified by Figure 1. On the representatively chosen area, single molecules and monoatomic steps of W(110) are simultaneously visible. The step edges appear as straight boarders of areas with almost constant color (the terraces) in the background of the image (Figure 1). On the terraces round bright structures are visible corresponding to the spherical shaped endohedral fullerenes. The image shows randomly distributed molecules on the W(110)-surface. Within our investigations, no preferred accumulation to favored adsorption positions like step edges have been observed. Individual molecules remain immobilized on the terraces and even attempts to induce self-assembling by surface diffusion via annealing (*T* = 670 K) were not successful. This means, the formation of monolayer islands is inhibited for this system. Apparently, the fullerene–W(110) bond is relatively strong. This is surprising in view of the well-known donation of six electrons from the incarcerated cluster to the cage [4], which causes a weaker adsorbate–substrate interaction [11] in comparison to empty fullerenes. The effects of higher annealing temperatures were not examined.

**Figure 2:**
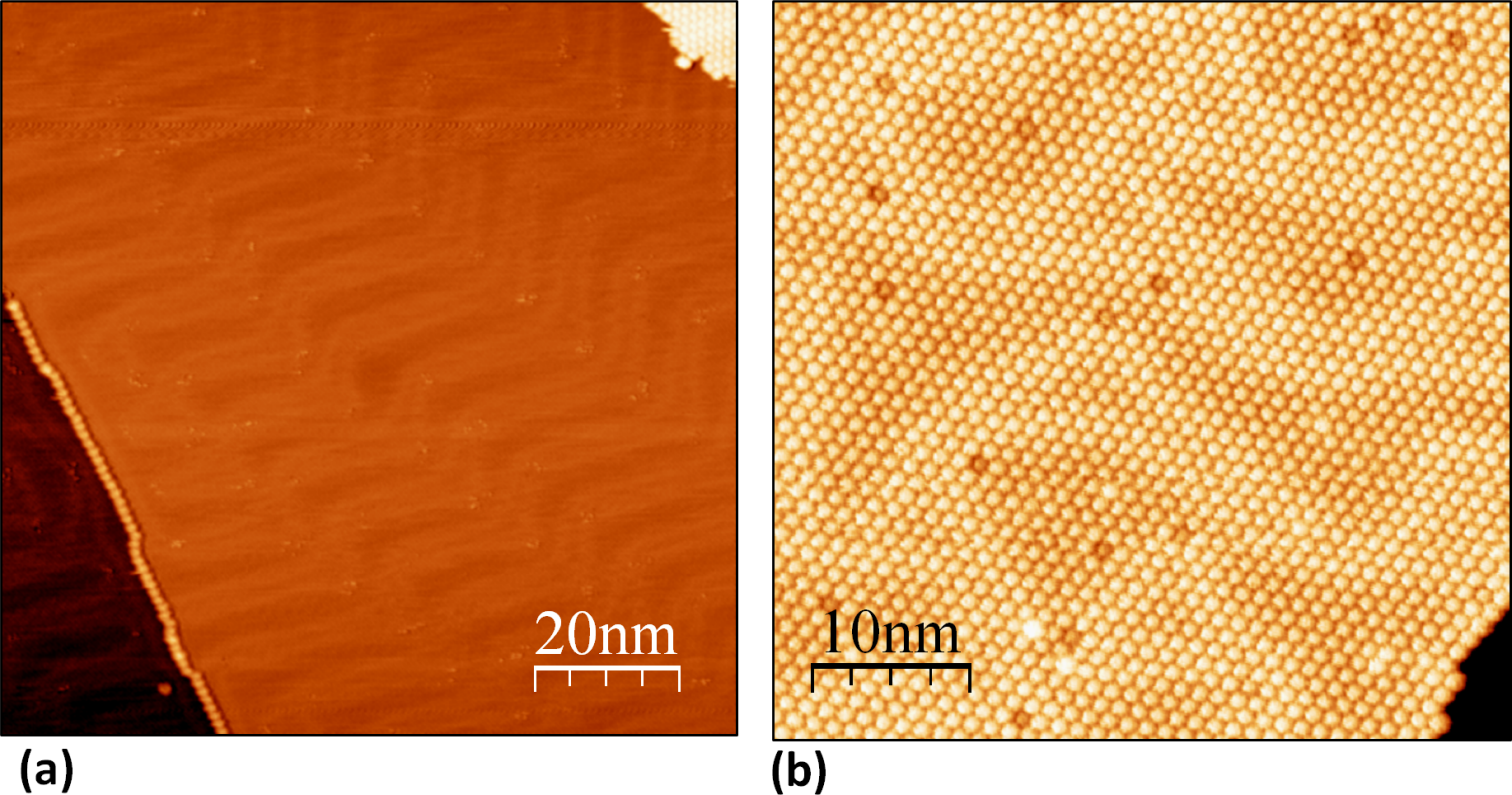
Topographic images (*U* = 1.5 V; *I* = 0.2 nA) of Er_3_N@C_80_ on Au(111). Figure (a) shows a one-dimensional chain of the endohedral fullerenes at the monoatomic step edge of the reconstructed Au(111) surface. Due to the presence of the molecules the reconstruction lines are distorted and prevented to pass the molecule line at the step edge. The molecules are positioned at fcc terminated adsorption sites indicated by the discommensuration line course. At the upper right corner the 2D-monolayer is visible. A fullerene monolayer on Au(111) is shown in Figure (b). The molecules arrange in a hcp-structure with a nearest neighbor distance on about 1.15 ± 0.01 nm. Several fullerenes of the monolayer appear darker respectively brighter in the image. This effect, caused by the fullerene monolayer induced restructuring of the Au(111) interface, could be assigned to an anomalous electronic structure resulting from the proposed formation of nanopits at the Au(111) interface that consequently lead to a changed number of Au-atoms interacting with the affected molecules [12,14]. Note that the presence of impurities cannot be excluded.

In contrast to the adsorption characteristics of Er_3_N@C_80_ on W(110), the molecules exhibit a sufficiently high mobility on the Au(111)-surface. In this case annealing initiated surface diffusion of the fullerenes on the terraces and along the lower level of step edges. Thus, they could form 1D single molecule lines at step edges (Figure 2a). These molecular lines seem to play an important role as initial nucleus for the 2D-growth of Er_3_N@C_80_ islands (Figure 2a,b), since at all observed instances, the 2D-islands are connected to step edges (not shown).

Note that under our preparation conditions fully saturated step edges were no precondition for the island growth to occur. Rarely, islands and inordinate aggregations on terraces were found where most likely local impurities served as nucleation points. The spatial extent of the monolayers reached sizes of several 100 × 100 nm^2^. The STM image of Figure 2b taken at the edge of an Er_3_N@C_80_-island illustrates that the molecules are organized in a hcp structure with a nearest neighbor distance of 1.15 ± 0.01 nm, consistent with earlier findings [11–12].

**Figure 3:**
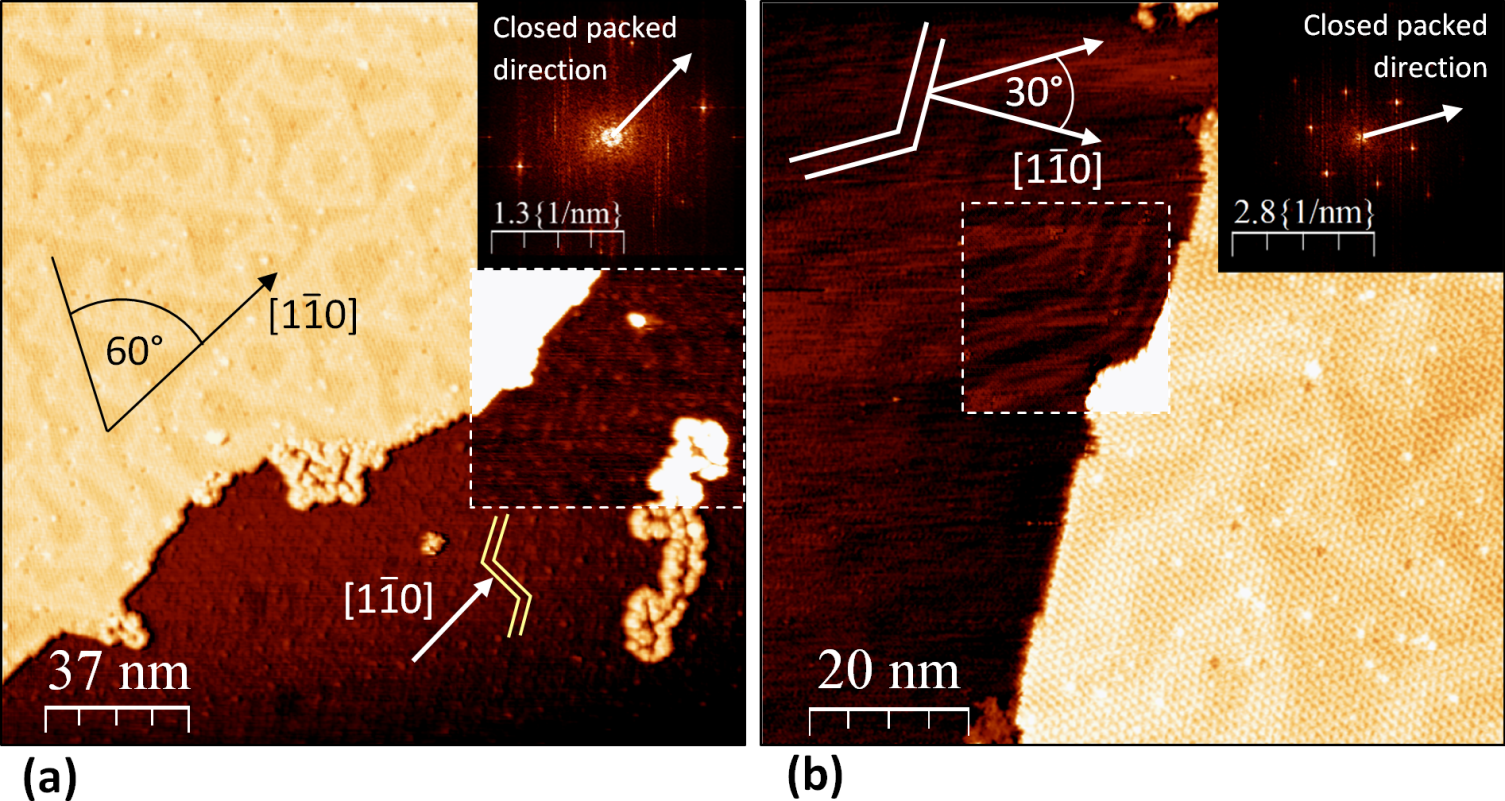
The Er_3_N@C_80_-monolayer orientations on Au(111) and the new interfacial reconstruction are depicted on these constant-current-images (*U* = 1.5 V; *I* = 0.2 nA). In Figure (a) the monolayer with in-phase orientation can be seen at the left upper half of the image. The arrows on the real-space image visualize the 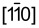-direction determined by the highlighted herringbone reconstruction (changed-contrast-inset and yellow double-line). The closed packed direction of the fullerenes is shown by an arrow inside the FFT-image (upper right corner) and corresponds to the Au(111)-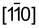-direction. The double line pattern of the interfacial reconstruction is visible on the monolayer. The pattern is dominated by ubiquitous 60°-angles. Figure (b) shows the out-of-phase oriented Er_3_N@C_80_-monolayer (right half) on Au(111). The closed packed direction of the molecules reveals a 30°-angle to the 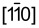-direction as described by the arrows inside the FFT-image (upper right corner) and the real space topography.

The orientation of the monolayers was examined by comparing them with the herringbone reconstruction visible at bare Au(111) surface regions (Figure 2a and Figure 3a,b). The typical double line pattern results from a 4.34% uniaxial compression along the closed packed <
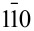
>-direction and runs perpendicular to that [15]. With respect to the non-reconstructed Au(111)-(1 × 1) surface, the orientation of the molecular adlayers closed-packed-direction coincides with Au(111)-<
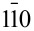
> (Figure 3a), whereby the monolayers can be described as (4 × 4) superstructure [11–12]. Our data reveal a new alignment in addition to this known in-phase orientation of Er_3_N@C_80_-monolayer on Au(111). In this case, the monolayer was found to be rotated to form an incommensurate (4 × 4)*R*30° phase (Figure 3b) with respect to the bulk fcc termination. Independent of monolayer orientation, the spacing between the fullerenes is the same value. This finding is reminiscent of results by Altman and Colton [16–17]. They performed detailed studies regarding the adsorption behavior of C_60_ on Au(111). There, even though the diameter of C_60_ is smaller than that of Er_3_N@C_80_, similar orientations of the monolayer were observed. Since the monolayer orientation of C_60_ is determined by the orientation of the step edge [16], a similar reason for the two observed phases of Er_3_N@C_80_-monolayers is likely. Thus, an adsorption to a step edge that is orientated along the [110]-direction would lead to an in phase monolayer.

Another interesting observation concerns the Au(111) surface reconstruction at the interface regions. As is evident from Figure 2a,b and Figure 3a,b, the reconstruction is modified by the interaction of the molecules with the bare Au(111)-surface. Beside the assembled fullerenes the herringbone reconstruction is visible at the bare Au(111) sections (Figure 2a and Figure 3a,b) as well as at the interface region (Figure 2a,b and Figure 3a,b). Due to the presence of the molecules, the herringbones are apparently disturbed and lose the straight long range zigzag course. This is the case for both, the one-dimensional molecular lines at the step edges as well as for the two-dimensional molecular layers. At the step edges (Figure 2a) the herring bones apparently cannot pass the single fullerene line. This is in contrast to the usual case of a clean Au(111) surface step edge where the reconstruction lines continue over the step edge [18]. Since the molecules are preferably located at the herringbone reconstruction’s fcc termination in our data, this termination appears energetically favorable for the adsorption.

In the 2D-case, beneath the Er_3_N@C_80_ covered areas the herringbone reconstruction is modified (Figure 3a,b) as well. This interfacial reconstruction reveals as a superimposed double stripe pattern on the monolayers. In contrast to the reconstruction of clean Au(111), the line pairs frequently coalesce to form rounded triangular structures (Figure 3a) and rather exhibit a less sharply defined course than for the clean Au(111)-surface. Figure 3a and b show that the reconstruction-lines between the covered and bare Au(111) areas are not linked to each other. Corresponding to the threefold symmetry of the Au(111) surface, 3 equivalent directions dominate the discommensuration line propagation, which can be concluded by ubiquitous 60° angles, as illustrated in Figure 3a. Thus, the elongation of the line pairs concerns the <
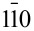
>-directions, the remaining compression of the Au-interface is indicated to point along <
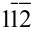
>. As compared to the spacing of 6.3 nm [15] between the line-pairs in the pristine surface, an increased and variable spacing (9.5–16 nm) was observed. These characteristics could be attributed to a decompression in the first atomic layer of Au(111) at the interface regions, which is likely induced by a rearrangement of Au-atoms in order to reduce interfacial energy. It seems plausible that reducing the mismatch between the monolayer and the Au(111) plays a crucial role for the latter, thereby enhancing the tendency of the Er_3_N@C_80_-adlayer to grow quasi-epitaxially on the Au(111)-surface. According to our data, the decrease of compression rather enlarged the distance between the line pairs than between the lines of the pairs. A favored adsorption on fcc sites is therefore suggested.

The observed interfacial reconstruction line pattern exhibits a lack of long range periodicity and uniformity as assumed for a thermodynamically most favored arrangement. This could be assigned to a frozen structure resulting from too short annealing times which implies that the rearrangement of the interfacial Au-atoms is most probably a thermally assisted and time-dependent process. While effects of longer annealing times and different temperatures were not further elaborated within this study, our conjecture is corroborated by pertinent results for C_60_-fullerenes on Au(111) [14,17,19–21].

The above results show that the monolayer growth and the minimization of the interface energy lead to a clear change of the reconstruction of the Au(111) surface. Considering the fact that the surface reconstruction of the pristine Au(111) is driven by a remarkable energy gain on 20 meV per Au-atom [22], and that van der Waals bond molecular layers on Au(111) typically leave the herringbone reconstruction unchanged [23] our observation of a modification of the reconstruction implies a molecular substrate interaction stronger than typical van der Waals interaction. A certain degree of hybridization of the molecules and Au(111) surface electronic structure is therefore conjectured. In order to investigate this further, we performed scanning tunneling spectroscopy (STS) on the Er_3_N@C_80_-monolayer on Au(111).

**Figure 4:**
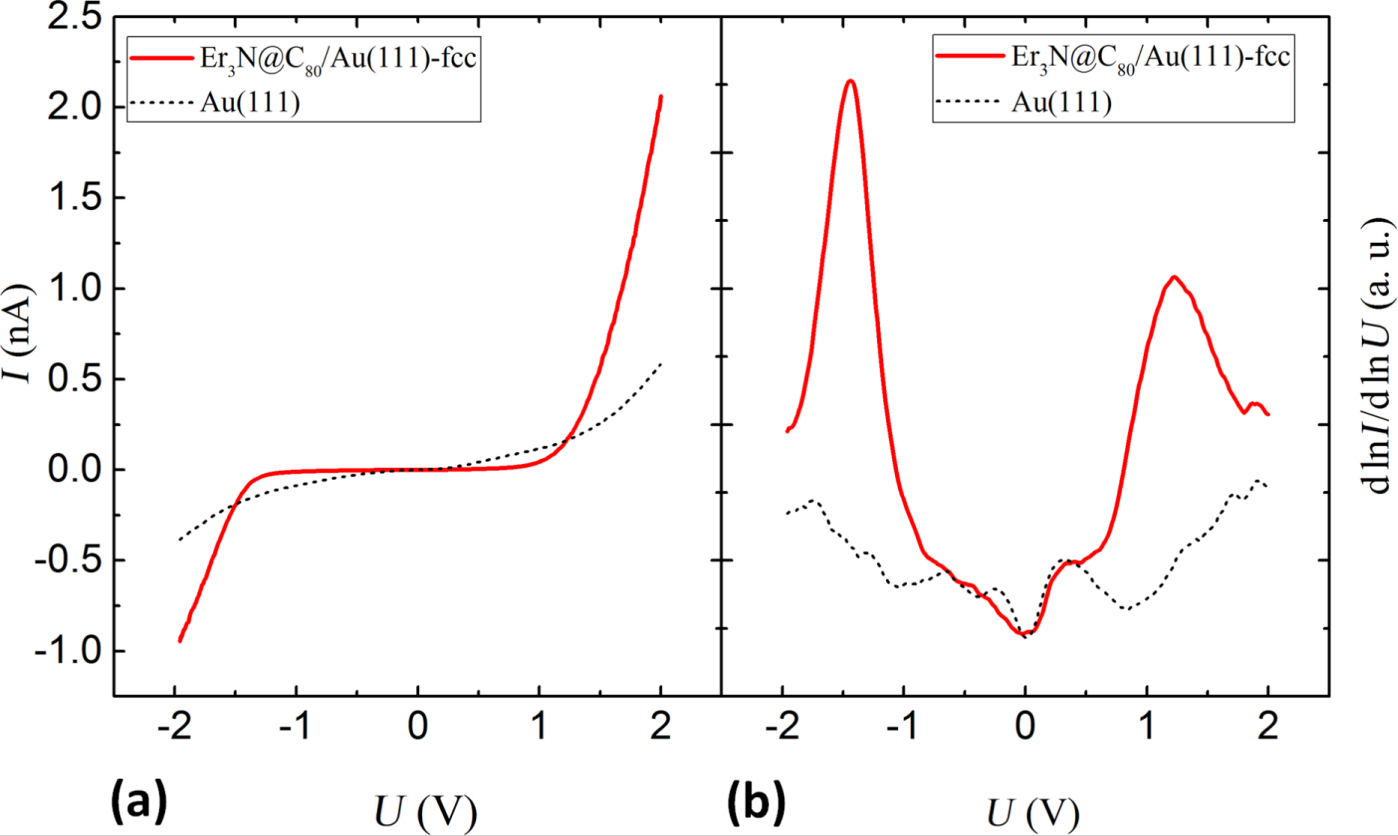
The *I*/*U*-spectrum (a) and the normalized dln*I*/dln*U*-spectrum (b) of Er_3_N@C_80_/Au(111). The voltage is considered as applied to the sample. The Au(111) surface state is not visible in the spectrum.

The obtained spectroscopic results, presented in Figure 4, exhibit two dominant peaks related to the HOMO- and LUMO-derived states (HDS and LDS) (Figure 4b). In between the molecular orbital derived states the tunneling current is suppressed (Figure 4a) and the HDS-LDS energy gap was determined to be of about 2.6 eV wide (peak to peak). The peaks are well defined and energetically located at a distinct distance to *E*_F_. This implies that even if a significant hybridization of molecular and substrate states occurs, the effective transfer of electrons remains relatively subtle. Nevertheless, the broadening of the peaks (≈1 eV) clearly exceeds the room temperature energy broadening (≈0.1 eV). Typical energies of intramolecular vibrations are also in the order of ≤0.2 eV.

It remains unclear, to what extent the additional broadening is due to a distribution of HOMO/LUMO multiplets or due to hybridization effects.

**Figure 5:**
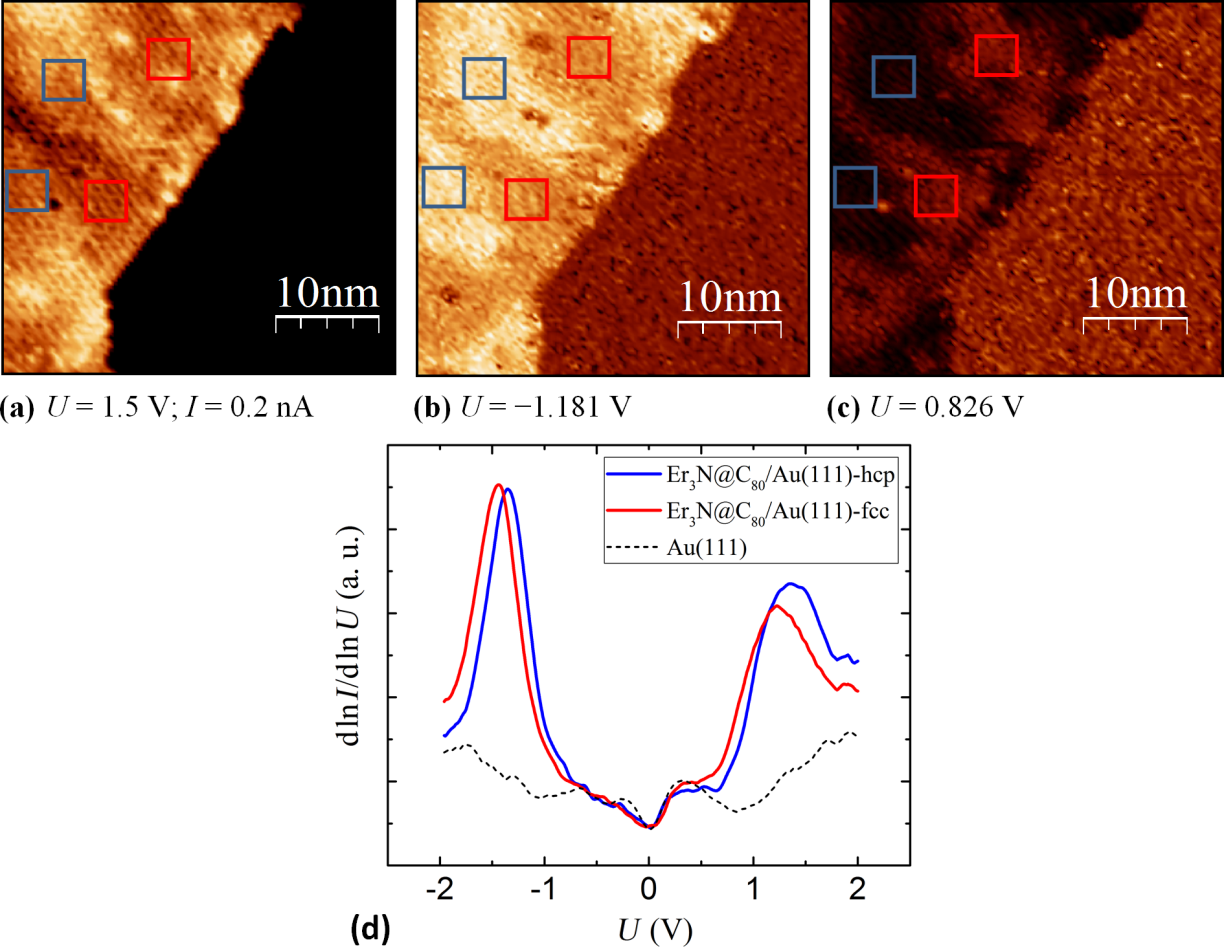
The left half of the constant-current-image (*U* = 1.5 V; *I* = 0.2 nA) (a) shows an Er_3_N@C_80_-monolayer and the pristine Au(111)-surface (right half). The corresponding d*I*/d*U*-maps at the voltages (considered as applied to the sample) of *U* = −1.181 V (b) and *U* = 0.826 V (c), reveal a bright dark pattern at the interface region accordingly to the interfacial reconstruction which is slightly visible in (a). The comparison of the dln*I*/dln*U*-spectra (d) (sample bias) taken at the highlighted (red respectively blue squares) areas in (a), (b) and (c), illustrate the spatial difference of the electronic structure.

In order to investigate this further, we performed spectroscopy d*I*/d*U*-mapping of the Er_3_N@C_80_-monolayers. Figure 5a shows the topographic data of the investigated area: the Er_3_N@C_80_-monolayer (bright half), the slightly visible interfacial reconstruction and the bare Au(111)-surface (dark half) can be seen. Interestingly, as illustrated in Figure 5b and c, spatial variations of the electronic structure occurred. The corresponding differential conductance maps (Figure 5b,c) respectively chosen at bias voltages of 0.826 V and −1.181 V reveal a bright-dark pattern on the monolayer coinciding with the interfacial reconstruction. Regions where the Au(111)-interface is considered to be of fcc termination appear darker in Figure 5b whereas the hcp adsorption sites are imaged brighter. Figure 5c shows the equivalent result with an inversed contrast. Since the fullerenes are located at distinguishable absorption sites, the observed contrast implies a spatially varying hybridization. This conclusion is supported by the comparison of the averaged spectra taken at the distinguished fcc and hcp areas (Figure 5d). Apparently, differences in the Au(111)-interface termination induce a 0.1 eV shift of the peaks relatively to each other. The features in the spectrum corresponding to the fcc regions (red) are positioned at lower energies relatively to *E*_F_ than those of the hcp related spectrum (blue). The rigid downward shift of the molecular derived states at the fcc adsorption sites could be assigned to a more pronounced pillow effect [24–26]. The influence of the interface dipole which appears stronger in the fcc regions led to a further reduction of the Au(111)-work-function accompanied by a reduction of the electron injection barrier. Furthermore, fcc-spectrums LUMO derived peak obviously exhibit a more pronounced broadening. Thus, a stronger adsorbate-substrate-interaction of the fullerenes on the fcc adsorption sites is suggested, leading to a higher degree of hybridization. This proposal could be consistent with a weaker bond of the Au-electrons in the fcc regions than that in hcp regions [27–28].

There is evidence that due to adsorption at fcc sites the Er_3_N@C_80_/Au(111) systems energy gain is the highest in comparison to the other possibilities. This interpretation is also consistent with the observed enlargement of fcc terminated region. Nevertheless, the energy gained by rearranging the interface Au-atoms in fcc termination is not high enough to entirely lift the reconstruction, i.e., the molecule-induced effect is competing with the energy gained by the typical contraction of the first atomic layer of the Au(111) surface (*E* = 20 meV/Au-atom [22]). It is conceivable that the observed Er_3_N@C_80_-monolayer on Au(111) did not reach its global energetically minimum and a change of the interfacial reconstruction pattern could occur in time.

## Conclusion

In the presented STM/STS-study, the respectively observed adsorption behavior of Er_3_N@C_80_ on W(110) and Au(111) is found to differ significantly from each other. On W(110) the endohedral fullerenes exhibits a surprisingly strong bond to the surface which inhibits monolayer formation via annealing in the analyzed temperature range (*T*_max_ ≈ 670 K). On the contrary to W(110), monolayer height molecule islands of hcp structure were formed on Au(111). Those monolayers possess two distinguishable orientations on the Au(111)-surface. Beside the known in-phase (4 × 4) superstructure an out-of-phase alignment (4 × 4)*R*30° has been observed. A change of the Au(111)-reconstruction initiated by the presence of the fullerenes indicates a energetically favored adsorption on fcc terminated Au(111) interface sites. The obtained STS data reveals a HDS-LDS-gap of about 2.6 eV and spatial differences in the energetic location of the peaks as well as of the LDS-peaks broadening on the monolayer. These results are consistent to our STM data, since the pattern of the found variations correlates with the modified reconstruction pattern and the spectra taken at adsorption sites of Au(111)-fcc termination are affected by means of an enhanced broadening of the LDS-peak and a shift of 0.1 eV towards lower energies. The obtained adsorption properties on Au(111) are namely the sufficiently high mobility to form island, the ability to modify the herringbone reconstruction and the hybridization of the molecular electronic states most probably accompanied with charge transfer. Therefore the bonding character is conjectured to exceed the strength of van der Waals interaction.
